# Biosafety evaluation of etoposide lipid nanomedicines in *C. elegans*

**DOI:** 10.1007/s13346-023-01466-w

**Published:** 2024-02-16

**Authors:** Souhaila H. El Moukhtari, Amanda Muñoz-Juan, Rubén Del Campo-Montoya, Anna Laromaine, María J. Blanco-Prieto

**Affiliations:** 1 Department of Pharmaceutical Technology and Chemistry, School of Pharmacy and Nutrition, Universidad de Navarra, C/Irunlarrea 1, 31008 Pamplona, Spain; 2 Instituto de Investigación Sanitaria de Navarra, IdiSNA, C/Irunlarrea 3, 31008 Pamplona, Spain; 3 Institut de Ciència de Materials de Barcelona (ICMAB-CSIC), Campus UAB, 08193 Bellaterra, Spain

**Keywords:** Etoposide, Lipid nanomedicines, Cancer, Neuroblastoma, *C. elegans*

## Abstract

Neuroblastoma is a pediatric tumor that originates during embryonic development and progresses into aggressive tumors, primarily affecting children under two years old. Many patients are diagnosed as high-risk and undergo chemotherapy, often leading to short- and long-term toxicities. Nanomedicine offers a promising solution to enhance drug efficacy and improve physical properties. In this study, lipid-based nanomedicines were developed with an average size of 140 nm, achieving a high encapsulation efficiency of over 90% for the anticancer drug etoposide. Then, cytotoxicity and apoptosis-inducing effects of these etoposide nanomedicines were assessed in vitro using human cell lines, both cancerous and non-cancerous. The results demonstrated that etoposide nanomedicines exhibited high toxicity and selectively induced apoptosis only in cancerous cells.

Next, the biosafety of these nanomedicines in *C. elegans*, a model organism, was evaluated by measuring survival, body size, and the effect on dividing cells. The findings showed that the nanomedicines had a safer profile than the free etoposide in this model. Notably, nanomedicines exerted etoposide’s antiproliferative effect only in highly proliferative germline cells. Therefore, the developed nanomedicines hold promise as safe drug delivery systems for etoposide, potentially leading to an improved therapeutic index for neuroblastoma treatment.

**Graphical Abstract:**

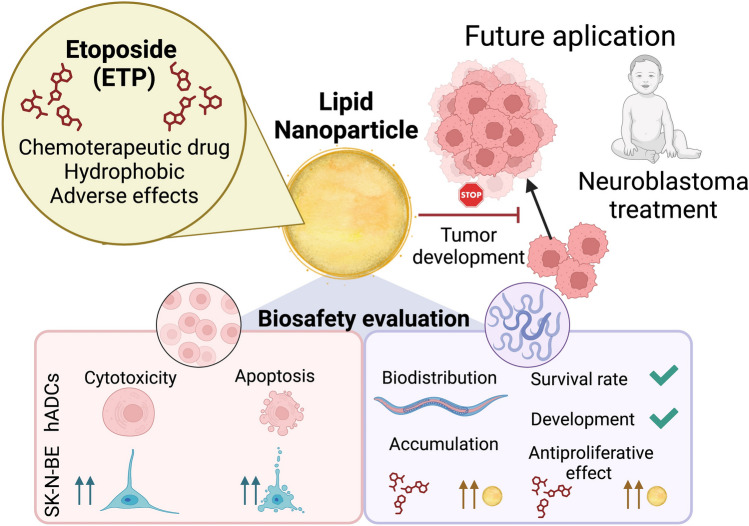

**Supplementary Information:**

The online version contains supplementary material available at 10.1007/s13346-023-01466-w.

## Introduction

Despite the numerous advances in cancer treatment in the last century, cancer remains one of the leading causes of death worldwide [1]. Pediatric cancers present a significant challenge due to the genetic diversity among these types of cancers, the fragility of pediatric populations, and the long life expectancy [2]. Neuroblastoma (NB) is an embryonal malignancy of the peripheral nervous system that usually develops in children under two years old and has a low prognosis [3]. Recent advances in NB treatment, such as antibodies and cell-based therapies, have improved some high-risk patients’ prognoses [4]. Chemotherapy remains a widely used treatment, although it can cause acute life-threatening effects and long-term toxicities affecting patients in adulthood [5]. In this context, nanomedicines have been demonstrated to improve the therapeutic index of drugs and also applied to deliver fragile molecules [6], theranostics [7], or immunotherapy [8].

Among chemotherapeutics, etoposide (ETP) has been widely used for various types of solid neoplasms as an oral and intravenous (iv) drug [9]. ETP is a phase-dependent topoisomerase II inhibitor derived from podophyllotoxin, which prevents cells from repairing DNA breaks during their cycle [10]. Despite this chemotherapeutic agent’s broad use, limitations have hindered its full potential. Moreover, the main dose-limiting toxicity is myelosuppression, which mostly manifests as leukopenia, although the mechanism has not been fully identified [11]. ETP also causes nausea and vomiting, but these side effects are more pronounced when the drug is administered orally. This drug also presents some long-term toxicities, including the development of secondary leukemias [11].

The long, prolonged administration of ETP generates a high cell cycle arrest effect against cancer cells [12]. However, therapeutic protocols usually employ short-term iv chemotherapy treatments. Oral ETP formulated in lipophilic soft capsules, known as Vepesid®, was initially designed for sustained drug release, but this treatment is associated with variable bioavailability, resulting in unpredictable behavior [13]. Additionally, ETP is poorly soluble in water and has limited stability in aqueous solutions. Thus, intravenously administered ETP requires complex formulations that include high amounts of polyethylene glycol, alcohols, and surfactants, leading to an increased risk of side effects.

Nanotechnology is a broad interdisciplinary field that aims for more specific diagnosis and treatments for an extensive range of diseases [14]. Specifically, nanoparticles (NPs) are becoming increasingly relevant for treating pediatric malignancies, with 55% of therapeutic molecules loaded into NPs for pediatric care being anticancer agents [15]. In particular, lipid NPs (LNPs) are known to efficiently improve the therapeutic index of hydrophobic molecules like ETP [16]. Moreover, LNPs have demonstrated effectiveness in oral administration for cancer treatment and enable lymphatic transport [17]. In our case, manufacturing these LNPs avoids the use of organic solvents, employing a sustainable, simple, and reproducible procedure. Previous in vitro cytotoxicity studies have demonstrated that these LNPs either maintained or improved the activity of ETP in cancer NB cells and unraveled their potential use in a combination therapy setting [18].

Over the last few years, awareness of the challenges faced in translating nanomedicines has increased [19, 20], highlighting the need to evaluate nanomaterials in multiple preclinical models and establish better connections between in vitro and in vivo models. *Caenorhabditis* *elegans* (*C. elegans*) was proposed as a model organism in 1965 [19], and has gained relevance in the nanotechnology field as a simple and reproducible model, offering a relevant physiological environment while adhering to the 3R rule [20, 21]. This model shares 60–80% of genetic homology with humans while sharing many fundamental biological processes [22]. ETP targets human topoisomerase IIα (topIIα) [10], which has two orthologous genes in *C. elegans*: *top-2* and *cin-4* [23]. Additionally, most of ETP metabolic routes are found in this model. Previous studies evaluated the effect of free ETP in *C. elegans* and demonstrated its impact on worm development, germ cells, and reproduction [23]. The model has also been used to study how a single change in one of the topIIα’s amino acids could affect the interaction with ETP. These unique characteristics, ease of growing large populations, simplicity, and transparency [20, 21], make this worm an attractive model for evaluating nanomedicines.

Taking into consideration all the aspects previously mentioned, the main objective of this study is to develop LNPs with ETP to reduce the drug’s toxicity and enhance its therapeutic index. Subsequently, the LNPs were characterized, and their cytotoxicity and apoptotic index were evaluated in human NB cells and healthy human adipose tissue-derived stem cells. In vitro experiments were complemented with in vivo experiments treating *C. elegans* with LNPs. Survival rate and body length were recorded at different time points, and the impact on the dividing cells of the model were measured. This LNPs proved to be safe drug delivery systems for ETP while improving its therapeutic index.

## Materials and methods

### Materials

For the NPs, ETP, rhodamine (ROD), and trehalose were acquired from Sigma Aldrich (Saint Louis, MO, USA), Precirol® ATO 5 was kindly provided by Gattefossé (Lyon, France), polysorbate 80 was purchased from Fagron (Terrassa, Spain). AMICON Ultra-15 10.000 MWCO filter devices were purchased from Merck Millipore (Darmstadt, Germany). Methanol (LC–MS) and chloroform were obtained from Scharlau (Sentmenat, Spain). For *C. elegans* maintenance, M9 buffer was utilized for bleaching, and K-medium was used for exposure, growth, and washing steps. In the reproduction assay, worms were incubated in nematode growth media (NGM). The compositions of the solutions used were as follows:

- M9 buffer: 5 g/L NaCl, 3 g/L KH_2_PO_4_ (both from Fisher), Na_2_HPO_4_, and 1 ml/L 1 M MgSO_4_ (both from Sigma Aldrich).
- K-medium: 10 ml/L 5 M NaCl, 30 ml/L 1 M KCl (Sigma), 3 ml/L 1 M CaCl_2_ (Labkem), 3 ml/L 1 M MgSO_4_, 240 µl/L cholesterol (5 mg/ml) (Sigma).
- NGM: 3 g/L NaCl, 1 ml/L 1 M MgSO_4_, 1 ml/L cholesterol, 1 ml/L CaCl_2_, 25 ml/L K_3_PO_4_ buffer, 17 g/L agar, 2.5 g/L peptone (last two from CONDA).
- K_3_PO_4_ buffer: 108 g/L KH_2_PO_4_ and 35.6 g/L K_2_HPO_4_ (Sigma).
  Paraformaldehyde (PFA) was purchased from Sigma.

## Formulation and characterization of etoposide nanoparticles

ETP-loaded nanoparticles (ETP-NPs) and unloaded NPs (Blank-NPs) were prepared using the hot homogenization and ultrasonication method, followed by freeze-drying for further studies, with slight modifications [24]. In brief, the lipid phase consisted of 300 mg of Precirol® ATO 5 with 15 mg ETP, while the aqueous phase consisted of 10 mL of a 5% (w/v) polysorbate 80 aqueous solution. The lipid phase was melted at 70 °C while the aqueous phase was heated at the same temperature. The aqueous solution was added to the lipid phase and mixed with an ultrasonic device for 4 min at 13 W. The resulting suspension was then cooled in an ice bath for 15 min and subjected to three washes with filtered water through diafiltration at 5000 g for 30 min to remove excess surfactant and non-incorporated drug using AMICON® Ultra-15 10.000 MWCO filter devices. Subsequently, ETP-NPs were suspended in an aqueous solution of trehalose (75% of trehalose/lipid) and freeze-dried. The hydrodynamic size and the polydispersity index (PDI) were determined in triplicate by dynamic light scattering (DLS), and zeta potential by laser Doppler velocimetry using the Smoluchowski equation with Zetasizer Nano (Malvern, UK). Size and homogeneity were also assessed by nanoparticle tracking analysis (NTA) with Nanosight (Malvern, UK). Each experiment was performed in triplicate, and all data are expressed as a mean value ± standard deviation (SD).

The amount of ETP entrapped into the NPs was determined using an ultra high-performance liquid chromatography–tandem mass spectrometry (UHPLC-MS/MS) method based on modifications of Gong et al. for mass spectrometry parameters [25]. Separation was conducted with an Acquity UHPLC™ BEH C18 PREMIER column (100 mm x 2.1 mm, 1.7 µm; Waters Corp., Milford MA, USA) with isocratic elution using a mobile phase composed of 50% of a 0.1% formic acid aqueous solution and 50% methanol. The column temperature was maintained at 50 °C, and the flow rate was set at 0.5 mL/min. The autosampler was conditioned at 4 °C, and the injection volume was 2 µL using partial loop mode for sample injection.

ETP was extracted from the NPs using a mixture of chloroform and methanol (1:2), followed by vortexing for 5 min and centrifugation at 12,500 g for 10 min. Subsequently, ETP was diluted in the mobile phase and quantified. For quantification, Triple-Quadrupole tandem mass spectrometric detection was performed on an Acquity™ TQD mass spectrometer (Waters Corp., Milford, MA, USA) with an electrospray ionization (ESI) interface. The mass spectrometer operated in positive mode and was set up for multiple reactions to monitor the transition of m/z 589.2 228.95. The following optimized MS parameters were employed: 1.5 kV capillary voltage, 20 V cone voltage, 120 °C source temperature, and 350 °C desolvation temperature. Results and data were analyzed using the Mass Lynx™ NT 4.1 Software with QuanLynxTM program (Waters Corp, Milford, MA, USA).

## Cell culture

SK-N-BE (2) cells were acquired from the ATCC (American Type Culture Collection) (Manassas, VI, USA). This cell line was cultured in Iscove’s Modified Dulbecco’s Medium, supplemented with 10% heat-inactivated fetal bovine serum, 1% of insulin-transferrin-selenium, and 100 U/mL penicillin/100 μg/mL streptomycin. For non-cancerous cells, hADCs (human adipose tissue-derived stem cells) cell line was used and kindly provided by the Clinic University of Navarra (Pamplona, Spain). This cell line was cultured in alpha minimum essential medium supplemented with 10% heat-inactivated fetal bovine serum, 1 ng/mL basic fibroblast growth factor, and 100 U/mL penicillin/100 μg/mL streptomycin. All reagents were obtained from Gibco, Thermo Fisher Scientific Inc., (Waltham, MA, USA). Both cell lines were cultured under 5% CO_2_ at 37 °C.

## In vitro cytotoxicity and apoptosis studies

The cytotoxicity of ETP and ETP-NPs was assessed using the MTS assay (PromegaCorp,USA) in SK-N-BE (2) cells and hADCs. For the SK-N-BE (2) cell line, 5000 cells per well were seeded in 96-well tissue culture plates, while for hADCs, 15,000 cells per well were seeded. The following day, cells were exposed to 20, 10, and 1 µM of equivalent dose of ETP for both free ETP and ETP-NP. Results are expressed as the mean data ± (SD) from at least three independent determinations.

The apoptotic activity of ETP-NP and ETP was determined in both cancerous (SK-N-BE (2)) and non-cancerous cell lines (hADCs), and the activity of caspase-3/7 was measured by Caspase-Glo-3/7 assay kit (PromegaCorp, USA) according to the manufacturer’s instructions. Briefly, cells were seeded at a density of 5000 cells per well for SK-N-BE (2) and 15,000 cells per well for hADCs in 96-well white plates. After 24 h, the cells were treated with ETP, ETP-NPs, blank-NPs, or control (media) and incubated for 72 h. Caspase activity was evaluated by adding the assay reagent to each well at a 1:1 ratio and measuring the luminescence signal in a Tecan GENios microplate reader (Tecan Group Ltd, Maennedorf, Switzerland) after 30 min of incubation. All samples were measured in triplicate, and the luminescence intensity was normalized to cell viability. Results are expressed as the mean data ± (SD) from at least three independent determinations.

## In vivo assessment in *C. elegans*

*C. elegans *Bristol N2 strain and *Escherichia coli* (*E. coli*) OP50 were obtained from Caenorhabditis Genetic Center stock collection (University of Minnesota, St. Paul, MN, USA). Worms were maintained at 20 °C, following previously described protocols [19]. Synchronization of worms at the L1 stage was achieved through the bleaching process [26]. In brief, a mixed population of worms was cleaned from bacteria and treated with 5 M NaOH (Sigma) and household bleach (ChemLab) (1:2) for 4 min in a final concentration of 1:10 to dissolve the worms’ tissues and keep eggs. The eggs were cleaned three times with M9 buffer and incubated 17 h at room temperature (RT) with gentle agitation for hatching. L1 worms were transferred to 96-well plates for exposure [27]. Each well contained approximately 15 ± 3 worms, paraformaldehyde-fixed *E. coli* OP50 (PFA-OP50) [28] in K-medium with an optical density at 600 nm of 0.75, and K-medium as a control, along with 100 µM free ETP, 100 µM of ETP-NP, and the equivalent dose of blank NPs, all resuspended in K-medium. Worms were exposed to each treatment for 72 h at 20°C (Fig. S1). ETP was initially resuspended in DMSO, and diluted in K-medium to a final percentage of 0.5% DMSO for *C. elegans* exposure. To avoid possible DMSO interferences, all the other conditions also had the same percentage of DMSO.

## Stability and drug release in *C. elegans* media

The stability and drug release of ETP-NP were assessed as preliminary tests before conducting the in vivo experiments in K-medium + OP50. NPs were added to *C. elegans* media under ETP’s sink conditions and incubated at 20 °C with gentle agitation. Samples were collected at various time points and analyzed to determine stability and drug release.

Regarding stability, the size and PDI were measured by DLS. For drug release assessment, samples were collected at specific time intervals, centrifuged at 5000 g with centrifugal filters, processed, and then diluted in the mobile phase for subsequent UHPLC-MS/MS analysis using the abovementioned conditions. The values are the mean ± SD of at least three independent determinations.

## Biodistribution studies in *C. elegans*

For the biodistribution assay, rhodamine (ROD) was encapsulated in the LNPs (ROD-NPs). The formulation method and freeze-drying process for ROD-NPs were identical to those used for ETP-NPs using the same preparation method. The worms were exposed to ROD-NPs, ETP-NPs, and Blank-NPs at the same concentrations. The amount of ROD encapsulated in LNPs was quantified by spectrofluorimetry at excitation (*λ*_ex_) and emission (*λ*_em_) wavelengths of 540 nm and 580 nm, respectively, using a Tecan GENios microplate reader (Tecan Group Ltd, Maennedorf, Switzerland). For comparison, worms were also treated with free ROD at a 9 µg/ml concentration to assess their biodistribution. After 72 h of exposure, worms were removed from the solution, washed, mounted on a microscope slide, and anesthetized in K-medium with 5 mM sodium azide (Sigma) for imaging. Imaging was performed using an Olympus BX51 optical microscope coupled to a fluorescent lamp. All images were acquired under the same light conditions to ensure consistency and accurate comparison of the biodistribution.

## Survival rate and body size

The survival rate and body size of worms were monitored every 24 h (Fig. S1). Worms were considered alive if they exhibited movement or responded to a physical stimulus [27]. A total of 180 worms were counted for each condition and replica. Data represent the mean ± standar error of the mean (SEM) of three independent experiments. To measure the body length [27], worms were washed by centrifugation for 1 min at 1300 g, the supernatant was removed, and fresh K-medium was added, repeating this process three times. Subsequently, the worms were incubated at RT for 2 h with 2% PFA and washed thrice using the same procedure. The worms were then mounted on microscope slides and imaged using an Olympus BX51 optical microscope. The obtained images were processed with ImageJ-Fiji Software. A total of 50 worms were measured for each condition and replica. Data represent the mean ± SD of three independent experiments.

## Etoposide internalization in worms

To measure the amount of ETP inside worms, L1 worms were transferred to 12-well plates for exposure. Each well contained approximately 500 worms, K-medium, PFA-fixed OP50, and either MilliQ water or 100 µM free ETP or 100 µM of equivalent dose of ETP for ETP-NP. After the exposure period, the worms were washed, and different fractions were separated, including the supernatant, washed fractions, and final pellet containing the worms. The amount of ETP accumulated in worms was then quantified by washing and drying the worms overnight at 60 °C. The resulting pellet was suspended in a methanol:water mix, followed by sonication with a sonication probe to extract the ETP from worms [21]. Samples were finally centrifuged at 12,500 g for 10 min, filtered, diluted in the mobile phase, and quantified using UHPLC-MS/MS.

## Reproduction index assay

After 72 h of exposure, worms were separated from the different solutions and individually transferred to NGM-plates with OP50. The worms were then maintained at 20 °C for an additional 72 h, during which the number of eggs and larvae was counted [27]. The primary objective of this study was to determine whether the antiproliferation effect of ETP persisted in the organs where it had accumulated.

To evaluate the antiproliferation effect of ETP, the reproduction index (RI) was calculated by dividing the total progeny number by the average progeny number of the control condition [27]. A RI higher than 1 indicates an increase in progeny (*i.e.,* cell proliferation), while a RI lower than 1 indicates a decrease in the progeny *(i.e.,* cell proliferation is inhibited). Each condition and replicate were assessed using six worms. Data represents mean ± SEM of three independent replicas.

## Statistical analysis

For cytotoxicity studies, an unpaired t-test was conducted. For apoptosis, survival, and reproduction assays, a one-way ANOVA test with Turkey’s multiple comparisons was used. The body size/length assay employed Kruskal–Wallis’s test with Dunn’s multiple comparisons. Each experiment was performed with at least three individual replicates. A significance level of p < 0.05 was considered statistically significant for all the conducted tests.

## Results and discussion

### Formulation and characterization of etoposide nanoparticles

**Fig. 1:**
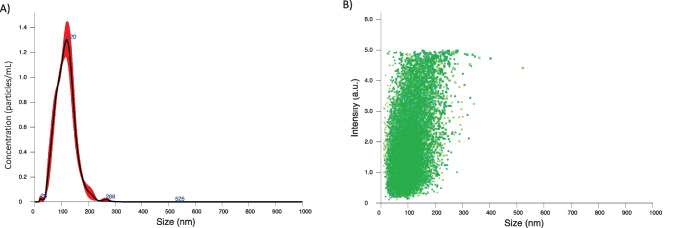
Nanoparticle Tracking Analysis using Nanosight®. Panel (**A**) shows the concentration of particles per milliliter (particles/mL) plotted against the size of ETP-NPs. Panel (**B**) depicts the representation of the scattered intensity of the intensity signal over the size of ETP-NPs

The results of size, PDI, and zeta potential obtained for the different types of developed LNPs are shown in Table S1. ETP-NPs showed a mean diameter of 140 ± 6.2 nm and homogenous distribution with a PDI value of 0.2 ± 0.1. These LNPs were negatively stabilized at -14 ± 4 mV, indicating an electrostatically stable surface charge (Table S1). Additionally, NTA revealed a homogeneous distribution of particles (Fig. 1A), with the intensity mainly concentrated around a particle size of 100 nm (Fig. 1B). ETP-NPs showed a high encapsulation efficiency (EE%) of approximately 91 ± 7.1%, resulting in a drug loading value of 22 ± 5.4 µg/mg. Those values indicate an optimization of the drug loading process compared to our previous results, where ETP-NPs exhibited an EE% of 75% [18]. The improvement was achieved by increasing the amount of ETP in the lipid phase and extending the dispersion time within the lipid. This higher drug-to-lipid ratio enhances biocompatibility in animal models and reduces the potential for toxic effects associated with more nanomaterial per kilogram of animal.

## In vitro cytotoxicity and apoptosis studies

Our previous studies [18] demonstrated that LNPs increased or maintained the cytotoxicity of ETP in different cancerous human NB cells. However, these studies focused solely on NB cells, and we were uncertain about the potentially toxic effects of ETP-NPs on healthy tissues. To address this concern, in vitro cytotoxicity assays on hADCs (healthy human cells) were conducted to determine whether the increased cytotoxicity observed in cancerous cells also occurred in healthy cells.

**Fig. 2:**
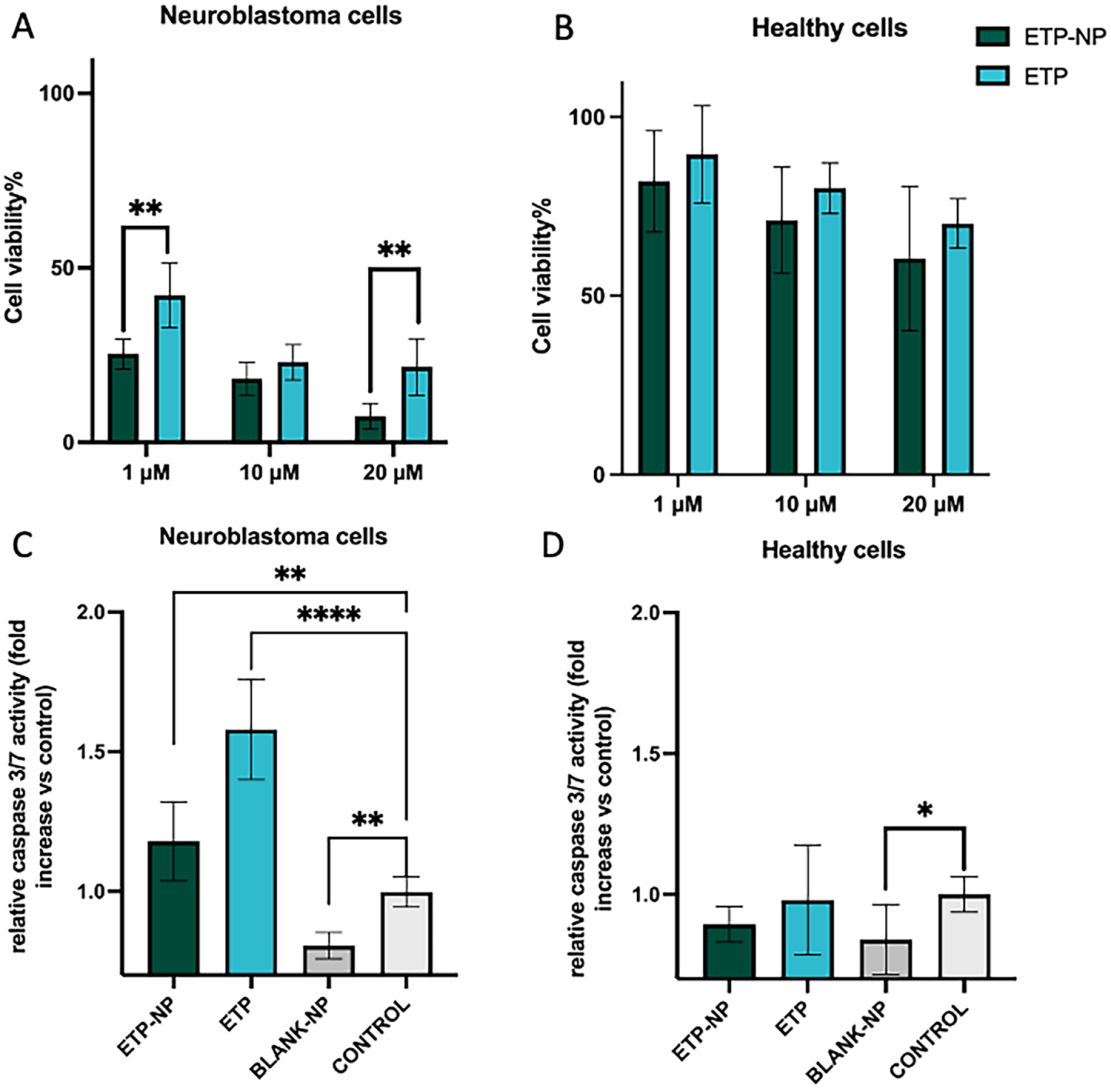
In vitro cytotoxicity of ETP and ETP-NPs in SK-N-BE(2) NB cells (**A**) and healthy hADCs (**B**) after 72 h of exposure at three different concentrations: high concentration (20 µM), medium concentration (10 µM), and low concentration (1 µM). Relative caspase 3/7 activity (fold increase vs. control) after the treatment of 20 µM of ETP, ETP-NPs, and the equivalent dose of blank-NPs in SK-N-BE(2) (**C**) and hADCs (**D**) at 72 h of exposure. Values are the mean ± standard deviation (SD) of at least three independent determinations, with triplicate values in each one

Results were compared with the effect found in NB cells, using SK-N-BE(2) cells as a control. For SK-N-BE(2) cells, we knew that encapsulating ETP reduced its IC_50_ from 0.752 ± 0.125 µM to 0.328 ± 0.150 µM. Figure 2 illustrates the cytotoxicity observed in hADCs and SK-N-BE(2) cells after 20 µM, 10 µM, and 1 µM ETP or ETP-NPs treatment following 72 h of exposure. Both ETP and ETP-NPs demonstrated higher efficacy in inhibiting NB cells than hADCs.

ETP and ETP-NPs demonstrated lethality in only approximately 30% of hADCs at high concentrations (20 µM). On the contrary, treating SK-N-BE(2) cells with ETP and ETP-NPs at the same concentration proved to be lethal in 80% and above 90% of these cells, respectively (Fig. 2A). It should be noted that hADCs exhibit lower sensitivity to ETP, primarily due to their higher doubling time when compared to SK-N-BE(2) cells. In contrast, other cell lines, such as fibroblasts with doubling times of around 30 h (data not shown), displayed higher sensitivity to ETP.

Moreover, in SK-N-BE(2) cells, statistical differences were found between ETP and ETP-NPs treatments at both high (20 µM) and low (1 µM) concentrations. On the other hand, for hADCs, no statistical differences were observed between ETP and ETP-NP treatments, indicating that nanoencapsulation does not increase toxicity in healthy cells. These results strongly suggest that LNPs could enhance the activity of ETP in NB tumors without increasing toxicity in healthy tissues. This promising finding indicates the potential of LNPs as a drug delivery strategy to improve the therapeutic efficacy of ETP while minimizing adverse effects on healthy tissues.

The apoptotic index was measured to confirm the selective cytotoxicity of ETP encapsulated (ETP-NPs) in SK-N-BE(2) cells while sparing healthy cells. ETP induces cell cycle arrest and apoptosis by promoting PKCδ and the caspase pathway in NB cells [31], although other routes have also been described [32, 33]. The apoptotic activity of the treatments was assessed by measuring caspase 3/7 activity after 72 h of exposure to 20 µM of ETP or ETP-NPs, as well as an equivalent dose of blank-NPs. The caspase 3/7 activity significantly increased after treatment with ETP and ETP-NPs in cancerous cells (Fig. 2C), confirming the findings from the cytotoxicity assays. Nevertheless, the apoptotic activity of free ETP appears to be higher than that of ETP-NP in NB cells. This phenomenon contrasts with what was observed in cell viability studies, but it can be explained by the fact that ETP induces cell death through both apoptosis and autophagy. In contrast, there was minimal apoptotic activity for both ETP-NP and ETP treatments in hADCs (Fig. 2D), confirming the safety of these NPs for healthy cells and suggesting their potential for safe in vivo administration. Regarding blank-NPs, the results indicate that caspase 3/7 activity is lower for blank-NP-treated cells than in the control and treatment groups (Fig. 2C, D). This demonstrates that unloaded NPs do not induce apoptosis, confirming that the employed nanomedicines are safe carriers without any apparent toxic activity.

## Stability and drug release in K-medium + OP50

**Fig. 3:**
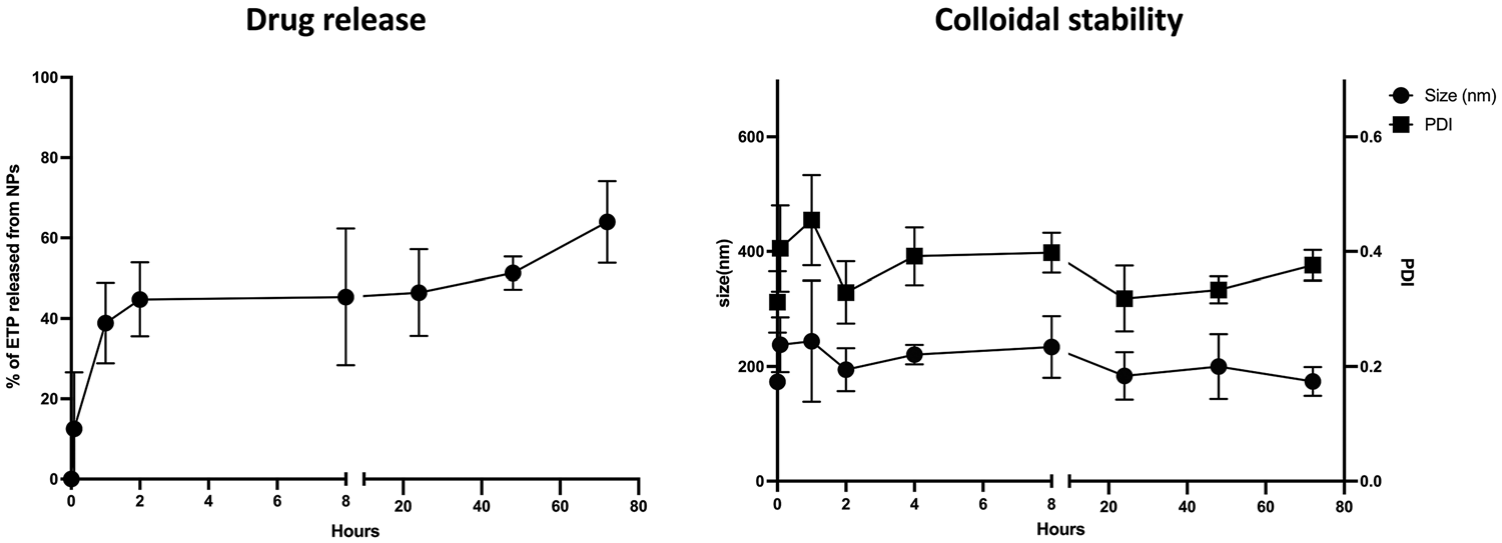
Stability and drug release of ETP-NPs in MK-OP50 media. Samples were taken from 0 to 72 h. The values are the mean ± standard deviation (SD) of at least three independent determinations

ETP-NPs stability and drug release were evaluated in K-medium supplemented with *Escherichia coli* (*E. coli*) OP50 (MK-OP50). This step was crucial as LNPs needed to remain stable in this media to ensure their ingestion by worms and for conducting biosafety assessments. ETP-NPs were added to MK-OP50 and taken under gentle and periodic agitation. Samples were obtained at 11 time points over 72 h, corresponding to the worms’ exposure time. The samples were characterized using DLS for size and PDI measurements, and UHPLC-MS/MS was employed to assess drug release. As shown in Fig. 3, LNPs demonstrated a controlled and prolonged release of ETP up to 60% at 72 h in this media. There was an initial burst release of 15% of ETP in the first few minutes after NPs were added to the worm media (K-medium). This could be explained by the composition of K-medium, which is rich in salts and contains cholesterol, favoring ETP’s solubility and release.

Regarding stability, we observed a noticeable increase in size and PDI from the first minutes of exposure up to the first hour, which stabilizes afterward. The size of ETP-NPs slightly increases from 143 ± 12 nm to around 200 nm, along with a minor increase in the PDI value, indicating no significant aggregation. Therefore, ETP-NPs remain stable in a saline media containing bacteria, which is a crucial factor for the oral administration of these LNPs, considering their interaction with the gastrointestinal microbiota for mammal absorption [29]. Furthermore, these physicochemical parameters are well-suited for evaluation in *C. elegans,* as they can efficiently uptake NPs below 500 nm [30]. Thus, ETP-NPs are stable in worm’s MK-OP50, as these nanocarriers do not aggregate and remain homogenous, suggesting that they can be effectively ingested by *C. elegans* orally*.*

## Biodistribution studies in *C. elegans*

To investigate the uptake and biodistribution of ETP-NPs in *C. elegans* fluorescent LNPs were prepared by encapsulating rhodamine (ROD) following the same formulation method. Briefly, ROD-NPs were added to *C. elegans* media for 72 h. As *C. elegans* ingest food by pumping liquid, we suspended ROD-NPs in MK-OP50 to simulate oral administration in these worms. The treatment was initiated at the early developmental stages (L1) and continued until adulthood.

**Fig. 4:**
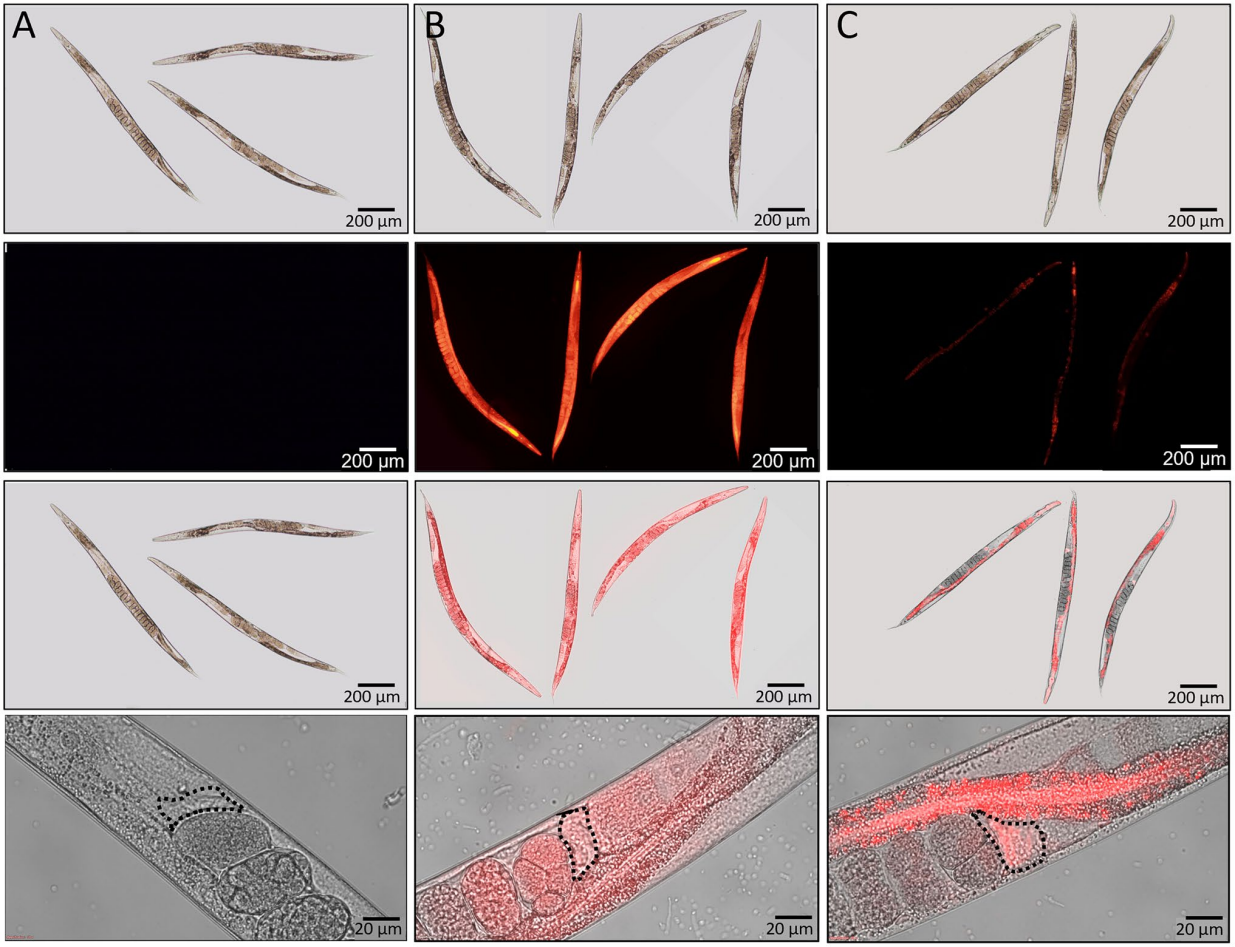
Biodistribution of ROD-NPs in *C. elegans*. Optical, fluorescent, and merged images of control (**A**), ROD-treated worms (**B**), and ROD-NPs-treated worms (**C**). Last row is a higher magnification than previous images and highlights one of the worms’ spermatheca

Control worms exposed solely to MK-OP50 were imaged to assess any potential interference of ROD with the autofluorescence of the worms. Additionally, we included a positive control group, with worms treated with the equivalent amount of free ROD to observe the biodistribution of this molecule before its encapsulation. As shown in Fig. 4A, control worms displayed a low level of autofluorescence, indicating minimal background signal. On the other hand, worms treated with free ROD exhibited complete staining with a red color (Fig. 4B), confirming the presence of the free ROD within the worms. This positive control enables us to distinguish the specific signal of encapsulated ROD-NPs from the inherent autofluorescence of the worms during subsequent biodistribution studies. All the *C. elegans* reached adulthood after 72 h.

After 72 h of exposure, ROD-NPs were detected in the intestinal tract of *C. elegans*, indicating that worms can orally ingest these LNPs (Fig. 4C). Interestingly, both worms’ spermathecas, localized at the end of each gonad, exhibited a higher red fluorescence intensity than other parts of the organism. This finding suggests that the encapsulated ROD successfully passes through the intestinal barrier and reaches specific reproductive structures within the worms.

## Biosafety evaluation in *C. elegans*

**Fig. 5:**
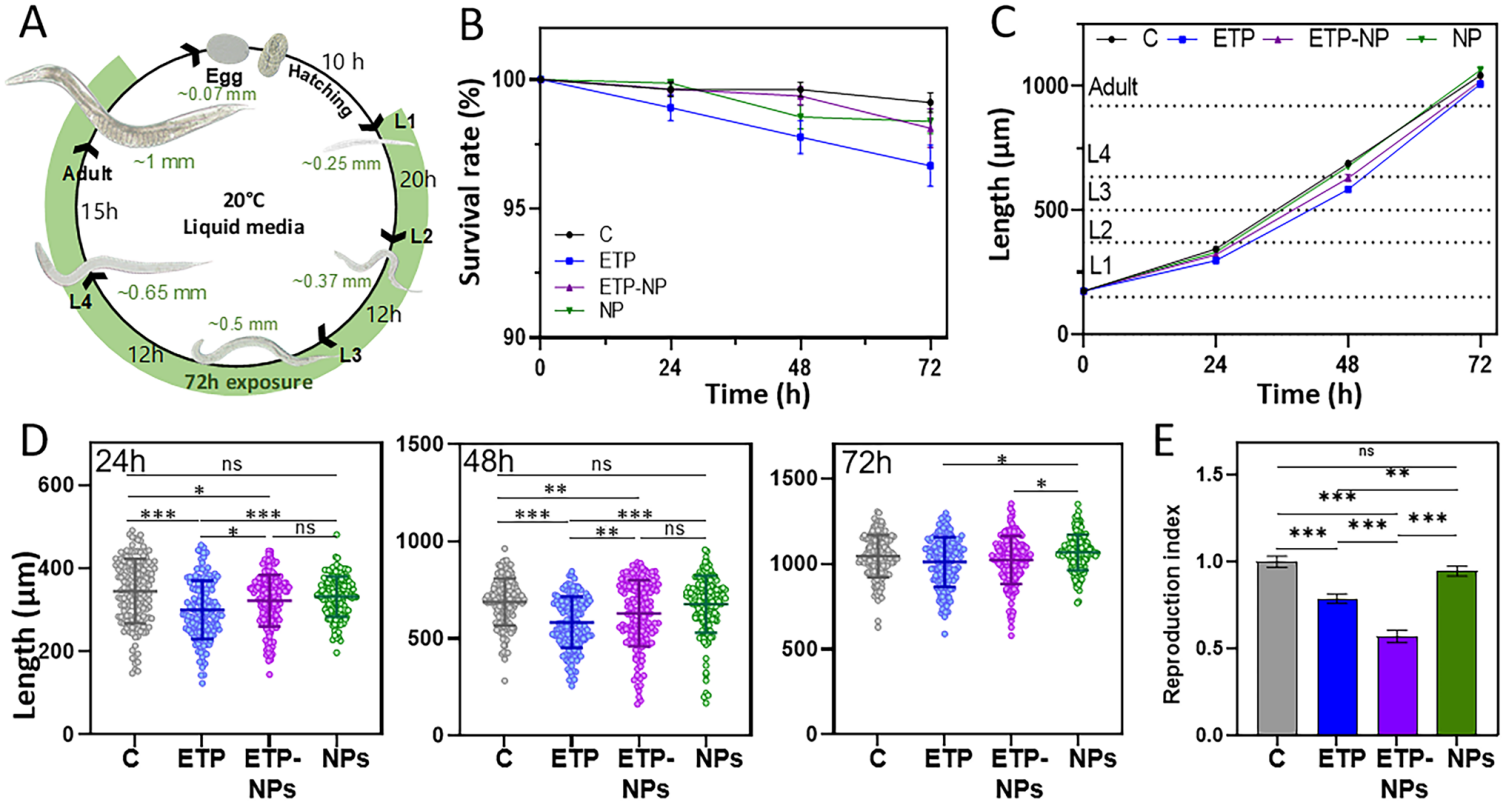
Biosafety evaluation in *C. elegans*. Worms’ life cycle at 20 °C showing the different developmental stages (**A**). Survival rate (**B**) and overall body length (**C**) after 72 h of exposure towards MilliQ water as a control (black), 100 µM ETP with (blue), 100 µM ETP-NPs (purple), and unloaded NPs (green). Data represents the mean ± standard deviation of the mean (SEM). Body length of each condition after 24, 48, and 72 h. Data represents mean ± standard deviation (SD) (**D**). Reproduction index of worms 72 h after stopping the treatment of the four previous conditions (**E**). Data represents the mean ± SEM. *p < 0.05, **p < 0.01, ***p < 0.001

After establishing that *C. elegans* could uptake LNPs, the biosafety of free ETP, ETP-NPs, and blank-NPs in this model was evaluated. Worms were treated from the L1 stage with 100 µM of each substance and a control group treated with MilliQ water. Thanks to the rapid *C. elegans* life cycle, which is completed in three days (Fig. 5A) [31], the effects of the drug for long treatment, from early larvae to adults could be observed (Fig. S1) [32]. The survival rate of the worms was monitored every 24 h over the course of the experiment. Remarkably, the survival rate of worms exposed to all tested substances remained higher than 95% (Fig. 5B); indicating no lethal effect at this concentration, consistent with previous findings [23]. Furthermore, the blank NPs did not negatively impact the worms’ survival rate, further confirming the excellent biocompatibility of these LNPs.

The worms‘ body length was closely monitored throughout the 72-h treatment. The results revealed that worms treated with free ETP significantly reduced body length, measuring 46 ± 25 µm shorter (** p < 0.01) than those treated with ETP-NPs. Additionally, both free ETP and ETP-NP treated groups were significantly smaller than the control group, measuring 105 ± 21 (*** p < 0.001) and 59 ± 24 (* p < 0.05) µm smaller, respectively, during the initial 48 h of treatment (Fig. 5C, D). However, after 72 h of treatment, no significant differences among the groups were observed, indicating that all conditions developed until adulthood. These results suggest that both ETP and ETP-NPs delay worms’ development during the initial stages, starting from L1 when the treatment commences. The encapsulation of ETP into LNPs effectively reduced the toxic effect observed with free ETP, as ETP-NP treated worms were significantly larger than ETP-treated worms during the first 48h, but they were still shorter than control worms. Moreover, no significant differences were found between the control worms and those treated with unloaded NP-treated, confirming the safety of these type of carriers in the *C. elegans* model.

Cell divisions in *C. elegans* occur less frequently compared to other model organisms. At the beginning of the L1 stage, worms have 556 cells, increasing by 403 more somatic cells in adulthood [33]. While ETP could potentially interfere with cell division through topoisomerase enzymes, previous studies have demonstrated that it primarily slows down worms’ development without affecting somatic cell division but impacts the worm’s germline [23, 34, 35]. Considering the increased accumulation of ROD-NPs in the worms spermatheca, we decided to complement our studies by evaluating the impact of ETP on the final offspring of treated worms.

At the L3/L4 stage (Fig. 5A), proximal germ cells in *C. elegans* enter meiosis [36] to form sperm, and later on, germ cells differentiate into oocytes. In adulthood, the germ cells are the only cells that continue dividing. After 72 h of treatment with ETP or ETP-NPs and another 72 h in fresh OP50-seeded NGM plates, we measured the total brood size (progeny) and calculated the reproductive index (Fig. 5E). The results revealed that treatment with free ETP led to a decrease of 21 ± 6% (** p < 0.01) in the reproductive index, while treatment with ETP-NPs caused a significant decline of 43 ± 7% (***p < 0.001). On the other hand, no toxic effects were found in worms treated with blank NPs. The biodistribution and reproductive index studies suggest that the NPs may be accumulating in the spermatheca, where they could sustainably release ETP over time, leading to a higher accumulation of ETP in worms’ spermatheca.

To confirm this, we extracted ETP from the worms after 72 h of treatment using a previously published method [21], and quantified it by UHPLC-MS/MS. The results indicated that drug uptake in worms was slightly higher for ETP-NPs at 0.24 ± 0.10% compared to 0.12 ± 0.09% for free ETP, although this difference was not statistically significant. These findings suggest that the encapsulation of ETP improves its bioavailability, resulting in higher inhibition of the germline compared to the free drug. The observed inhibition of cell proliferation in the germline appears to be directly related to the accumulation of NPs in the spermatheca of worms. Since germline cells are the only cells actively dividing in adulthood, they seem more affected by ETP than somatic cells in *C. elegans* [37]. The precise reason for this selective impact on germline cells is unclear [35], but previous works suggested that carcinogenic drugs like ETP or cisplatin activate DNA damage response pathways specifically in the worm germline but not in other cells [35].

## Conclusions

Our study successfully developed ETP-NPs using the hot homogenization and ultrasonication method, obtaining homogenous LNPs with a narrow size distribution. The encapsulation protocol was optimized, resulting in increased encapsulation efficiency. Previous experiments showed enhanced ETP efficacy of these LNPs in cancerous NB cell lines. In this study, we demonstrated the safety of these nanomedicines in vitro with healthy cell lines and in vivo with the *C. elegans* model.

The toxicity of ETP and ETP-NPs was higher in cancerous cells than in healthy cells. Importantly, nanoencapsulation increased the lethality of ETP, specifically in NB cells, while sparing healthy cells. This selective increase in antiproliferative activity only in cancer cells suggests that these LNPs can serve as safe carriers for ETP, enhancing its effectiveness in treating cancer.

In the *C. elegans* model, ETP-NPs were localized along the intestinal tract and crossed the intestinal barrier, accumulating in the spermatheca. Their administration proved safe based on the survival and body size of the worms, showing ETP-NPs had a lower impact on worms’ development than free ETP. The presence of ETP-NPs in the spermatheca increased the accumulation of ETP in the region where the adult worm’s dividing cells (germline cells) are located, reducing the worm’s reproduction index. The nanoencapsulation of ETP increased its activity in dividing cells while having little impact on quiescent cells (rest of the worm), consistent with in vitro results with healthy and NB cells.

In light of these findings, we conclude that these LNPs offer a promising approach for delivering ETP, as they enhance its therapeutic index while ensuring safety. The selective increase in antiproliferative activity in cancer cells suggests that these LNPs have the potential to improve the effectiveness of ETP in cancer treatments, offering a safer and more efficient therapeutic option for patients with neuroblastoma. Future research will concentrate on optimizing the administration of ETP-NPs in more complex in vivo models, evaluating their effectiveness in treating neuroblastoma.

### Supplementary Information

Below is the link to the electronic supplementary material.

Supplementary file1 (DOCX 153 KB)

## Data Availability

The data and materials of this study are available on reasonable request.
