# Correction: Biosafety evaluation of etoposide lipid nanomedicines in *C. elegans*

**DOI:** 10.1007/s13346-024-01568-z

**Published:** 2024-03-08

**Authors:** Souhaila H. El Moukhtari, Amanda Muñoz-Juan, Rubén Del Campo-Montoya, Anna Laromaine, María J. Blanco-Prieto

**Affiliations:** 1 Department of Pharmaceutical Technology and Chemistry, School of Pharmacy and Nutrition, Universidad de Navarra, C/Irunlarrea 1, 31008 Pamplona, Spain; 2 Instituto de Investigación Sanitaria de Navarra, IdiSNA, C/Irunlarrea 3, 31008 Pamplona, Spain; 3 Institut de Ciència de Materials de Barcelona (ICMAB-CSIC), Campus UAB, 08193 Bellaterra, Spain

**Correction: Drug Delivery and Translational Research**

10.1007/s13346-023-01466-w

In the original online version of this article the following author note was missing: Souhaila H. El Moukhtari and Amanda Muñoz-Juan are first co-authors and contributed equally to this work.

The original article was corrected.

