# Supplementary material for: Biosafety evaluation of etoposide lipid nanomedicines in C. elegans
Source: Drug Deliv Transl Res. 2024 Feb 16;14(8):2158–69. doi: 10.1007/s13346-023-01466-w (PMC11208201; doi:10.1007/s13346-023-01466-w)
Supplement: Supplementary file 1 — Supplementary file1 (DOCX 153 KB) [file 13346_2023_1466_MOESM1_ESM.docx]

**SUPPLEMENTARY INFORMATION**


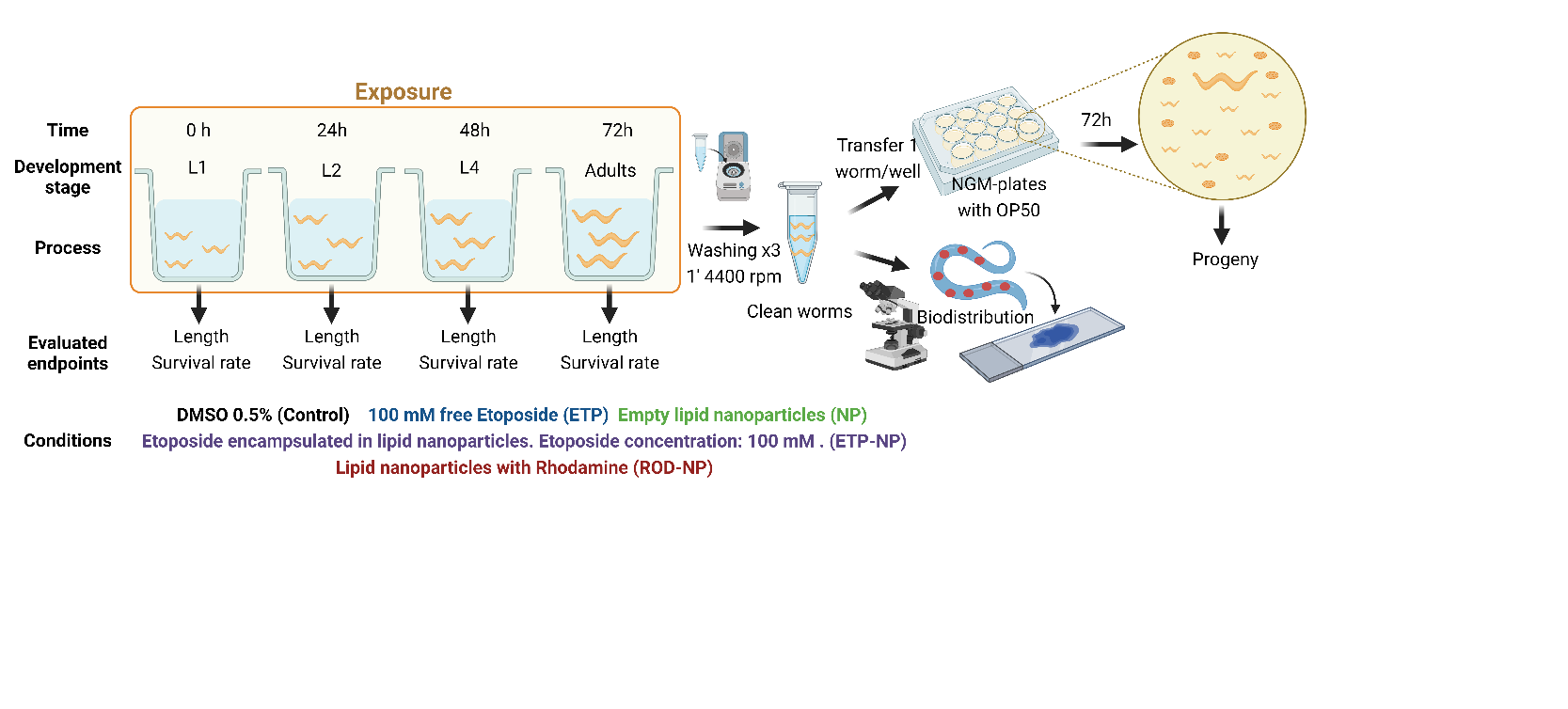


**Figure S1**. Experimental setup for the exposure to LNPs and toxicity endpoints checked in this study.

**Table S1.** Physicochemical characteristics of unloaded nanoparticles (Blank-NP), etoposide loaded nanoparticles (ETP-NP), and rhodamine loaded nanoparticles (ROD-NP). Values are the mean ± standard deviation (SD) of at least three independent determinations.

|  | Size | PDI | Z potential |
| --- | --- | --- | --- |
| BLANK-NP | 152 ± 7.13 nm | 0.215 ± 0.022 | - 15.1 ± 0.70 mV |
| ETP-NP | 140 ± 6.21 nm | 0.181 ± 0.112 | - 14.8 ± 4.34 mV |
| ROD-NP | 147 ± 12.13 nm | 0.194 ± 0.018 | - 6.18 ± 2.63 mV |
